# Effect of Morning Light Glasses and Night Short-Wavelength Filter Glasses on Sleep-Wake Rhythmicity in Medical Inpatients

**DOI:** 10.3389/fphys.2020.00005

**Published:** 2020-01-28

**Authors:** Chiara Formentin, Stefano Carraro, Matteo Turco, Lisa Zarantonello, Paolo Angeli, Sara Montagnese

**Affiliations:** Department of Medicine, University of Padua, Padua, Italy

**Keywords:** light therapy, glasses, entrainment, sleep-wake rhythm, filter

## Abstract

Sleep and circadian rhythm disorders are common amongst medical inpatients. They are caused by a mixture of factors, including noise, loss of habitual daily routines, and abnormal exposure to light, which tends to be insufficient in the day and too high at night. The aim of the present study was to test the efficacy of morning light therapy plus night short-wavelength filter glasses on sleep quality/timing, and sleepiness/mood over the daytime hours, in a group of well-characterized medical inpatients. Thirty-three inpatients were enrolled and randomized (2:1) to either treatment (*n* = 22; 13 males, 48.3 ± 13.3 years) or standard of care (*n* = 11; 8 males, 56.9 ± 12.9 years). On admission, all underwent a baseline assessment of sleep quality/timing and diurnal preference. During hospitalization they underwent monitoring of sleep quality/timing (sleep diaries and actigraphy), plus hourly assessment of sleepiness/mood during the daytime hours on one, standard day of hospitalization. Patients in the treatment arm were administered bright light through glasses immediately after awakening, and wore short-wavelength filter glasses in the evening hours. Treated and untreated patients were comparable in terms of demographics, disease severity/comorbidity, diurnal preference and pre-admission sleep quality/timing. During hospitalization, sleep diaries documented a trend for a lower number of night awakenings in treated compared to untreated patients (1.6 ± 0.8 vs. 2.4 ± 1.3, *p* = 0.057). Actigraphy documented significantly earlier day mode in treated compared to untreated patients (06:39 ± 00:35 vs. 07:44 ± 00:40, *p* = 0.008). Sleepiness during a standard day of hospitalization, recorded between 09:30 and 21:30, showed physiological variation in treated compared to untreated patients, who exhibited a more blunted profile. The level of sleepiness reported by treated patients was lower over the 09:30–14:30 interval, i.e., soon after light administration (interaction effect: *F* = 2.661; *p* = 0.026). Mood levels were generally higher in treated patients, with statistically significant differences over the 09:30–14:30 time interval, i.e., soon after light administration (treatment: *F* = 5.692, *p* = 0.026). In conclusion, treatment with morning bright light and short-wavelength filter glasses in the evening, which was well tolerated, showed positive results in terms of sleepiness/mood over the morning hours and a trend for decreased night awakenings.

## Introduction

Sleep-wake disturbances are common in hospitalized patients (Tranmer et al., 2003; Humphries, 2008; Missildine et al., 2010). Insomnia derives from both intrinsic, endogenous factors (i.e., physical illness and pain, psychological stress) and unfavorable, exogenous environmental stimuli. Loss of habitual daily routines, fixed schedules, therapeutic and diagnostic procedures are some of the main reasons for sleep interruptions and poor sleep quality (Freedman et al., 2001; van Kamp and Davies, 2008; Kamdar et al., 2012). Patients are removed from their familiar setting and placed in a new environment, which may result in disorientation for time and space, especially in elderly patients, and altered circadian rhythmicity.

Hospital life is very disruptive also in terms of wake quality, as patients spend a significant amount of time in bed, and are rarely capable and/or provided with adequate mobilization. In turn, spending too much time in bed and being inactive during the day can lead to so-called learnt insomnia, and to the prescription of sleep-inducing medication (Spielman et al., 2005).

Abnormal exposure to light, which tends to be too low in the day and too high at night (Friese, 2008; Bano et al., 2014; Bernhofer et al., 2014) may also contribute to sleep-wake disturbance of circadian origin, as light is the main environmental cue (*Zeitgeber*) for synchronizing the circadian clock to the environment. In health-care facilities, low light exposure levels and a reduced difference between day and night environmental lighting conditions may alter rhythmicity, and thus contribute to the impairment in sleep quality (Meyer et al., 1994; Kamdar et al., 2012).

Based on the above observations, the aim of the present study was to test the efficacy of morning light therapy, in combination with night short-wavelength filter glasses, on sleep quality/timing and the time course of sleepiness/mood in a group of well-characterized medical inpatients. The duration of treatment was that of hospitalization.

## Patients and Methods

On admission, all patients enrolled in the study underwent an assessment of their pre-hospitalization sleep quality/timing, and were then randomized to either the treatment or the control group, by random numbers generated from a computer. They were asked to complete a sleep diary every morning and to wear a Jawbone actiwatch for the whole duration of their hospitalization. In addition, they were asked to choose a single day for hourly completion of the Karolinska Sleepiness Scale (KSS; *vide infra*) and a 1–10 Visual Analogue Scale (VAS) of mood, from get up time to bedtime. Patients in the treatment arm were also asked to wear light glasses in the morning (*vide infra*) and short-wavelength filter glasses from 18:00 h (*vide infra*). Except for sources of environmental disturbance due to hospital routines, patients were free to go to bed/try to sleep and wake/get up at their chosen times. Spontaneous night awakenings were unlikely to result in lights on, while awakenings related to admissions/hospital routines were likely to result in lights on. Patients in the treatment arm were instructed to wear their short-wavelength filter glasses in the latter instance.

### Patients

Forty-five patients admitted into the medical ward Clinica Medica V of Padua University Hospital between February 2016 and July 2018 were screened. Ten were unwilling to take part in the study and two were subsequently excluded for poor compliance with the study protocol. Thus the final population included 33 inpatients (51 ± 14 years) randomized (2:1) to either treatment (*n* = 22; 48 ± 15 years) or standard of care (*n* = 11; 58 ± 5 years).

The majority of patients were hospitalized for liver/biliary/pancreatic diseases (42%), this being due to the fact that the unit also serves as a tertiary referral hepatology center. Other diagnoses on admission were cardiovascular diseases (34%), infections (including pneumonia, urinary tract infection and erysipelas; 12%), and gastrointestinal bleeding or acute anemia (12%).

The Charlson Comorbidity Index was used for purposes of assessment of disease severity/comorbidity. This ranges from 0 to 37, and a weight is assigned to each of 19 medical conditions, based on the corresponding relative risk of death within 12 months (Charlson et al., 1987).

Exclusion criteria were: age >80 years, severe cognitive impairment, inability to adhere to the protocol/comply with the tasks, lack of informed consent, diagnosed sleep–wake disorders (i.e., obstructive sleep apnoea, restless legs syndrome etc.), shift work or intercontinental travel over the preceding 6 months.

Over the inpatient stay, data were collected daily on the administration of sleep-inducing (benzodiazepines or benzodiazepine-like) and other psychoactive drugs (antidepressants, neuroleptics, and opioid analgesics), with the idea that patients could benefit from light-dark treatment in terms of insomnia/sleep problems, thus limiting the need for pharmacological intervention.

The study protocol was approved by the local Ethics Committee and the study conducted according to the Declaration of Helsinki (Hong Kong Amendment) and Good Clinical Practice (European) guidelines. All participants provided written informed consent.

### Sleep-Wake Assessment

On the first day of hospitalization, all participants underwent an assessment of their baseline, pre-hospitalization sleep quality and timing, based on the following questionnaires/scales:

• The Pittsburgh Sleep Quality Index (PSQI), which evaluates subjective sleep quality over the preceding month, and differentiates “good” from “poor” sleepers. Responses to the 24 questions of this self-administered questionnaire are used to generate seven components, each of which is scored from 0 to 3 (0 = best). The PSQI total score is the sum of all domains (range 0–21), and a total score >5 characterizes “poor sleepers” (Buysse et al., 1989; Curcio et al., 2013). Component 4, which allows obtainment of average get up and bedtime data, was used for purposes of assessment of pre-hospitalization sleep timing.
• The Horne–Östberg (HÖ) questionnaire, which defines diurnal preference as definitely morning (score 70–86), moderately morning (59–69), intermediate (42–58), moderately evening (31–41), and definitely evening (16–30) based on 19 self-administered questions (Horne and Ostberg, 1976).
• The KSS, a self-rated subjective sleepiness scale (range 1: “very alert” – 9: “very sleepy, fighting sleep, difficulty staying awake”) (Akerstedt and Gillberg, 1990), which patients were asked to complete hourly (from wake up to sleep onset time) on a standard day of hospitalization, except from hospitalization day 1. This is because the first day of hospitalization often follows a sleepless night, a prolonged stay in the Accident and Emergency area and a generally disrupted routine.
• A VAS of mood (range 1: worst to 10: best); this was also completed hourly (from wake up to sleep onset time) on a standard day of hospitalization, except from hospitalization day 1.

During the whole period of hospitalization, patients were asked to:

• Complete a sleep diary daily, recording bedtime, sleep onset, time taken to fall asleep, wake up time, get up time, and the number and duration of any night awakenings and/or daytime naps. Night awakenings were intended as either spontaneous arousals or sleep interruptions due to environmental factors. Sleep-onset latency was calculated as the difference between bedtime and sleep onset time (in minutes); time spent in bed as the difference between bedtime and get up time (in hours); length of sleep as the difference between sleep onset and wake up time (in hours). Patients were asked to complete their sleep diaries early in the morning, immediately after waking up, in order to limit recall bias, as they could not generally take notes during the night. Each diary page also included a VAS for the assessment of sleep quality during the previous night (range 1: worst to 10: best) (Lockley et al., 1999; Carney et al., 2012);
• Wear a Jawbone^TM^ UP24 (Jawbone, San Francisco, CA, United States), i.e., a bracelet type wrist-worn device which records movement by use of an accelerometer. The simple assumption underlying the technique is wake = movement, sleep = lack of movement (Cellini et al., 2013). Data were then downloaded and analyzed by the pertinent application UP by Jawbone^TM^. The following indices were obtained: time spent in bed, total sleep (i.e., total duration of all epochs of sleep during period of time in bed), sleep latency [(i.e., the difference between “night mode,” *vide infra*, and the first epoch qualified as sleep (Cook et al., 2018)], number of awakenings, day mode [i.e., time in the morning, after wake up time, when the patient pressed a button on the actiwatch band to switch from “sleep mode” to “active mode”; this function is a so-called “event marker,” a strategy to enhance the quality of information obtained from actigraphy (Martin and Hakim, 2011)], night mode (i.e., time at night, after bedtime, when the patient pressed the same button to switch from “active mode” to “sleep mode”).

### Light-Dark Treatment Schedule

**FIGURE 1:**
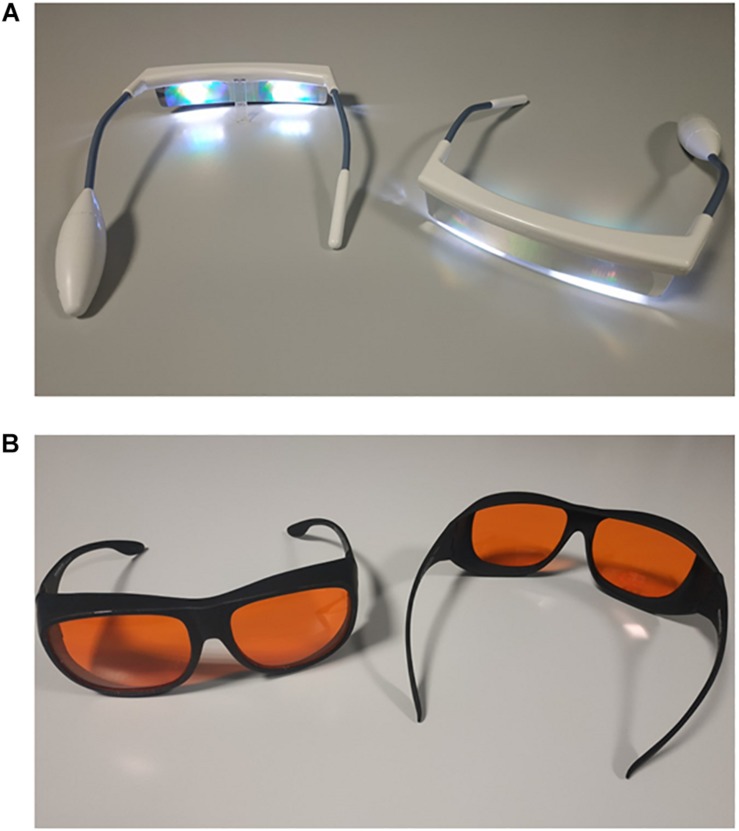
Luminette light glasses [Lucimed, Villers-le-Bouillet, Belgium; **(A)**] with light emitting diodes (approximately 2000 lux; blue-enriched 400–750 nm) and short-wavelength filter glasses [MelaMedic, Viborg, Denmark; **(B)**], which filter light in the blue range of the spectrum, i.e., the one our visual circadian timing system is most sensitive to.

Patients in the treatment arm were provided with light glasses (Figure 1A) and short-wavelength filter glasses (Figure 1B). They were instructed to wear their light glasses for 30 min in the morning, after waking up (i.e., while having breakfast, immediately after their personal hygiene, etc.) and to wear their short-wavelength filter glasses from 18:00 h until sleep onset time, plus at any time overnight when the light in their room was on. Exact timings were patient-driven and both types of glasses were managed individually by the patients (i.e., not distributed and/or operated by staff at any given time).

### Equipment

• Luminette^®^ light glasses (Langevin et al., 2014) (Lucimed, Villers-le-Bouillet, Belgium; Figure 1A), which can be worn over prescription glasses, are equipped with eight Light Emitting Diodes (LEDs) distributed on the upper part of the two lenses (four on each side), outside the patient’s visual field. The LEDs reflect light (2000 lux; blue-enriched 400–750 nm) toward the eye via a diffractive lens, thus focusing light toward the lower part of the retina, regardless of the position of the head. This also allows for uniform illumination and for dazzling avoidance (Bragard and Coucke, 2013).
• Short-wavelength filter glasses (MelaMedic, Viborg, Denmark; Figure 1B), which can be worn over prescription glasses, filter light in the blue range of the spectrum, i.e., the one our visual circadian timing system is most sensitive to (Foster, 2005). Thus they limit exposure to awakening light signals at times when the natural environment is dark but the artificial one inside the hospital is well lit or the patients use mobile phones, tablets, reading devices which emit relatively strong and blue-enriched light (Gringras et al., 2015).

Illuminance levels at night, as measured in a previous study performed by our research group in the same medical ward, were 12 ± 24 lux between 23:30 and 00:30 (Bano et al., 2014). Information on the variability of both day and night lighting conditions, plus noise levels is also provided in the same study (Bano et al., 2014).

### Statistical Analyses

Data are presented as mean ± SD or median and range, as appropriate. The distribution of variables was tested for normality using the Shapiro–Wilk’s *W*-test. Differences between normally and non-normally distributed variables were evaluated by the Student *t* or Mann Whitney *U* test, respectively. Categorical variables were compared by the Pearson’s χ^2^ or Fisher’s exact test, as appropriate. The time-course of subjective sleepiness/mood was analyzed by repeated measures ANOVA, by treatment group. After analyzing the whole period 09:30–21:30, analysis was performed also on the 09:30–14:30 interval (*post hoc*, unplanned). Analyses were also repeated after missing data had been imputed by the k-nearest neighbor method. Correlations between sleep diaries and actigraphic variables were tested by the Pearson *r*.

## Results

### Baseline

Treated and untreated patients were comparable in terms of demographic characteristics (Table 1). Seven patients slept in single rooms (five treated and two untreated), four in double rooms (two treated and two untreated), 22 in quadruple rooms (15 treated and 7 untreated). Nineteen patients (13 treated and 6 untreated) were qualified as sleeping near (distance < 1 m) and 14 (9 treated and 5 untreated) as sleeping far (distance > 3 m) from the room window. The average length of hospitalization was 4 (range 3–12) days. No significant differences were observed between treated and untreated patients in terms of distribution in single/double/quadruple rooms, position in relation to the window or average length of hospitalization [4 (range 3–12) vs. 3 (range 3–12) days, n.s.; Table 1]. The Charlson Comorbidity Index was also comparable in the two groups [1 (range 0–6) vs. 2 (range 0–5), n.s.; Table 1].

**TABLE 1:** Patients’ features, by treatment group.

**Features**		**Total (*n* = 33)**	**Treated (*n* = 22)**	**Untreated (*n* = 11)**	***p***	**t/*U*/χ/*OR***
Demographics	Age (years; mean ± SD)	51.1 ± 13.6	48.3 ± 13.3	56.9 ± 12.9	0.086	−1.774
	Gender (% males)	64	59	73	0.355	*0.542*
	Charlson Comorbidity Index (median; range)	2 (0–6)	1 (0–6)	2 (0–5)	0.158	*83.5*
Hospitalization	Room type (single/double/quadruple, n)	7/4/22	5/2/15	2/2/7	0.743	0.594
	Bed position (near/far from the window, n)	19/14	13/9	6/5	0.547	*0.831*
	Length of hospitalization (days; median; range)	4 (3–12)	4 (3–12)	3 (3–12)	0.909	*117.5*
Diurnal preference and	HÖ (mean ± SD)	56.6 ± 7.4	56.8 ± 6.7	56.1 ± 9.5	0.841	0.203
Night sleep quality	PSQI (mean ± SD)	6.0 ± 3.2	6.0 ± 3.1	5.9 ± 3.7	0.931	0.088

*HÖ, Horne–Östberg morningness–eveningness questionnaire; PSQI, Pittsburgh Sleep Quality Index.*

Diurnal preference and pre-admission sleep quality were comparable in treated and untreated patients (HÖ: 56.8 ± 6.7 vs. 56.1 ± 9.5, n.s.; PSQI: 6.0 ± 3.1 vs. 5.9 ± 3.7, n.s.). Pre-admission sleep timing, as summarized in component 4 of the PSQI, was also comparable (get up time: 06:43 ± 01:11 vs. 07:01 ± 00:48, n.s.; bedtime 23:14 ± 00:44 vs. 23:26 ± 01:22, n.s.).

### Inpatient Stay

Daily sleep diaries were regularly obtained for the whole length of hospitalization for all patients. By contrast, due to poor compliance (i.e., several patients did not wear the Jawbone regularly) and technical issues (depleted battery), only an average 3-day actigraphy recordings (first 3 days of hospitalization) per patient were obtained and analyzable. Actigraphic data were available for 18 patients for day 4, 13 patients for day 5, 10 patients for day 6, and 6–2 patients for days 7–12. There were no significant differences between compliant (actigraphy for the full length of hospitalization) and non-compliant (actigraphy for no more than 3 days) patients in terms of age, Charlson Comorbidity Index, PSQI, HÖ and length of hospitalization.

Diaries documented a trend for a lower number of night awakenings in treated compared to untreated patients (1.65 ± 0.82 vs. 2.42 ± 1.26, *p* = 0.057; Table 2). All other diary parameters were comparable in the two groups (Table 2). Actigraphy documented significantly earlier day mode switch time in treated compared to untreated patients (06:39 ± 00:35 vs. 07:44 ± 00:40, *p* = 0.008; Table 3). All other Jawbone parameters were comparable in the two groups (Table 3).

**TABLE 2:** Sleep-wake timing based on daily diaries (entire hospitalization period).

	**Treated, mean/*median* (n)**	**Untreated, mean/*median* (n)**	***p***	**t/*U* value**
Bedtime (hh:mm ± hh:min)	22:23 ± 00:43 (21)	22:16 ± 01:05 (9)	0.740	0.335
Time try to sleep (hh:mm ± hh:min)	23:01 ± 00:32 (21)	22:52 ± 00:58 (10)	0.623	0.497
Sleep onset (hh:mm ± hh:min)	23:22 ± 00:36 (21)	23:11 ± 00:59 (10)	0.552	0.601
Wake up time (hh:mm ± hh:min)	06:31 ± 00:44 (21)	06:20 ± 00:28 (9)	0.707	0.379
Get up time (hh:mm ± hh:min)	07:02 ± 00:45 (21)	07:01 ± 00:33 (8)	0.680	–0.417
Daytime naps (n)	0.25 (0–2.57) (21)	0.67 (0–1.08) (10)	0.272	*78.5*
Night awakenings (n)	1.65 ± 0.82* (21)	2.42 ± 1.26* (10)	0.057	–1.978
Sleep onset latency (min)	22 (3–45) (21)	15 (6–52) (10)	0.597	*92*
Length of sleep (h)	7.17 ± 0.77 (21)	6.87 ± 1.47 (10)	0.489	0.700
Time spent in bed (h)	8.5 (7.12–10.03) (21)	8.54 (5.37–10.44) (8)	0.733	*76.5*
Sleep quality (1–10)	5.78 ± 1.62 (20)	6.27 ± 1.57 (10)	0.574	–0.569

*p < 0.05.

**TABLE 3:** Actigraphic indices averaged over the first three days of hospitalization.

	**Treated (n)**	**Untreated (n)**	***p***	***t* value**
Total sleep (h)	6.57 ± 1.43 (13)	6.58 ± 0.93 (6)	0.970	−0.038
Sleep latency (h)	2.77 ± 0.48 (12)	1.93 ± 0.98 (6)	0.257	1.176
Time spent in bed (h)	9.20 ± 1.13 (12)	8.52 ± 0.97 (6)	0.253	1.185
Awakenings (n)	1.75 ± 0.85 (21)	2.37 ± 1.29 (10)	0.120	−1.601
Day mode (hh:mm ± min)	06:39 ± 00:35* (11)	07:44 ± 00:40* (5)	0.008	−3.084
Night mode (hh:mm ± min)	20:48 ± 02.38 (14)	21:58 ± 00:28 (5)	0.352	−0.958

*p < 0.05.

Comparing get up time before (Component 4 of PSQI) and during hospitalization (get up time on sleep diaries and day mode on actigraphy), by treatment group, no significant changes were observed over time.

Significant correlations were observed between a number of parallel diary- and actigraphy-derived parameters (i.e., number of awakenings, *r* = 0.96; *p* < 0.001; get up times/day mode, *r* = 0.53; *p* = 0.029).

**FIGURE 2:**
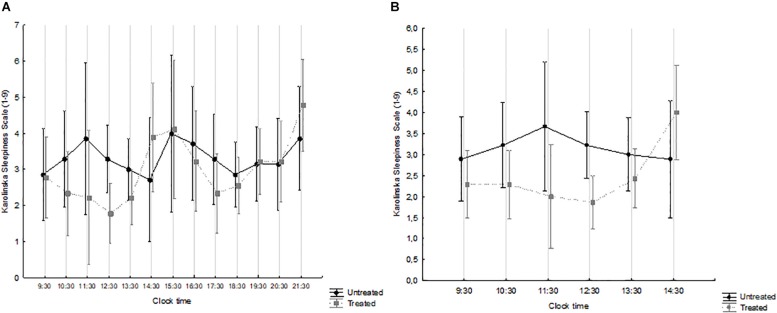
Karolinska Sleepiness Scale (KSS) scores, from 09:30 to 21:30 **(A)** and from 09:30 to 14:30 **(B)** in patients treated with Luminette^®^ light glasses plus short-wavelength filter glasses (gray squares and broken line) and untreated patients (black circles and full line). **(A)** Time: *F* = 1.932, *p* = 0.034; treatment: *F* = 0.481, *p* = 0.499; interaction time × treatment: *F* = 1.164, *p* = 0.313. treated *n* = 9; untreated *n* = 7. **(B)** Time: *F* = 1.210, *p* = 0.310; treatment: *F* = 2.342, *p* = 0.141; interaction time × treatment: *F* = 2.661, *p* = 0.026. treated *n* = 14; untreated *n* = 9.

The overall (waking hours) and the morning time-course of sleepiness are shown in Figures 2A and B, respectively, by treatment group. Over the waking hours (Figure 2A), the effect of time was significant (*F* = 1.932, *p* = 0.034), while that of treatment was not (*F* = 0.481, *p* = 0.499), and there was no interaction (*F* = 1.164, *p* = 0.313). By contrast, the levels of sleepiness reported by treated patients were significantly lower over the 09:30–14:30 interval, i.e., immediately after the course of light treatment (interaction effect: *F* = 2.661; *p* = 0.026) (Figure 2B). Overall, treated patients exhibited a considerably more physiological time-course of subjective sleepiness, which decreased during the morning, peaked in the early afternoon and then started increasing again in the early evening (Eriksen et al., 2005; Ekstedt et al., 2009; Turco et al., 2015).

**FIGURE 3:**
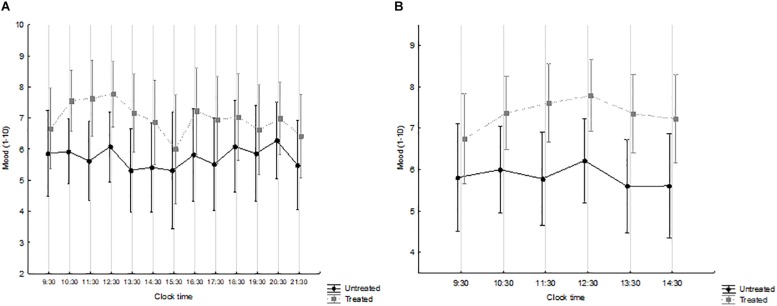
Mood (1–10 visual-analogue scale), from 09:30 to 21:30 **(A)** and from 09:30 to 14:30 **(B)** in patients treated with Luminette light glasses plus short-wavelength filter glasses (gray squares and broken line) and untreated patients (black circles and full line). **(A)** Time: *F* = 1.583, *p* = 0.100; treatment: *F* = 2.887, *p* = 0.110; interaction time × treatment: *F* = 0.719, *p* = 0.732. treated *n* = 9; untreated *n* = 7. **(B)** Time: *F* = 1.773, *p* = 0.124; treatment: *F* = 5.692, *p* = 0.026; interaction time × treatment: *F* = 0.698, *p* = 0.626. treated *n* = 14; untreated *n* = 9.

The overall (waking hours) and the morning time-course of mood are shown in Figures 3A and B, respectively, by treatment group. Mood levels were generally higher in treated patients. Over the waking hours (Figure 3A), no significant effects were observed. By contrast, over the morning hours, i.e., immediately after the course of light treatment (Figure 3B), time was not significant (*F* = 1.773, *p* = 0.124) while treatment was (*F* = 5.692, *p* = 0.026), with no interaction (*F* = 0.698, *p* = 0.626).

Results did not change when the time course of sleepiness and mood (over part of the day) was re-analyzed after missing data had been imputed by the k-nearest neighbor method.

Finally, sleep-inducing drugs were prescribed to three patients in the treatment group and four in the control group. Two of these patients, one per group, were already on sleep-inducing medication prior to hospitalization. When sleep-inducing medication use was expressed as a% (nights on sleep-inducing medication/total inpatient nights), no significant differences in the use of sleep-inducing drugs were observed between treated and untreated patients (10 ± 28 vs. 29 ± 46%, *p* = 0.152).

## Discussion

In hospitalized patients, the combination of disease, modified light, food and activity cues, together with sleeping within a noisy, unusual environment, results in altered circadian rhythmicity, poor sleep quality and increased number of night awakenings. Among the environmental/exogenous factors that influence sleep-wake rhythmicity, inefficient photic resetting of the circadian timing system plays a powerful role within the “arrhythmic” hospital environment, and seems to be a promising field for intervention (Bano et al., 2014). In the present study, we used a combination of short-wavelength filter glasses plus a 30-min course of light administration in a group of well-characterized medical inpatients, with the aim of guaranteeing darkness at night and providing higher amounts of strong, blue-enriched light in the early morning, as an entrainment cue. Treated patients showed a trend for a lower number of night awakenings on sleep diaries and earlier wake up time/day mode on actigraphy records compared to their untreated counterparts. While compliance with actigraphy was limited and a number of technical problems were encountered, correlations were detected between parallel diary and actigraphic parameters. Moreover, in treated patients, the time course of subjective sleepiness over the waking hours was closer to the physiological one (Eriksen et al., 2005; Ekstedt et al., 2009; Turco et al., 2015), and mood was also better. This is in line with a significant amount of literature on the positive effects of light on mood (Wirz-Justice et al., 2005), which have been rarely, however, tested in inpatients. Finally, while statistical significance was not reached, sleep-inducing medication was more commonly prescribed in untreated compared to treated patients. This observation, together with the earlier day mode switch and better mood observed in treated patients, is extremely interesting from a clinical stand point, as a more efficient performance over the early waking hours may have positive domino effects, making medical inpatients more amenable and more responsive to other entrainment cues, such as food intake and physical activity (i.e., if the patient is found to be sleepy in the early morning rounds, he/she is much more likely to skip breakfast and/or a physiotherapy session).

Over recent years, evidence has emerged that abnormalities in circadian regulation (misalignment), altered sleep-wake cycles and poor sleep quality have serious medical consequences, including an increased incidence of diabetes, obesity, metabolic syndrome (Spiegel et al., 2009), cardio-vascular accidents (Zhong et al., 2005; Haupt et al., 2008; Sauvet et al., 2010; Vetter et al., 2016), and even certain types of cancer (Masri and Sassone-Corsi, 2018; Walasa et al., 2018). It is therefore possible to hypothesize that misalignment, and especially hospitalization-related misalignment, may have a further, negative impact in patients who already have these conditions, making countermeasures akin to those used in this study clinically relevant. Larger, most likely multicenter studies are needed to address this issue.

Overall, compliance with both types of “glasses” was good: only two patients did not regularly use them and were subsequently excluded for poor adherence with the protocol. In general, both types of glasses were well tolerated and seemed comfortable to wear, also on top of prescription glasses. While wearing them, patients were free to perform their usual activities (for example have breakfast, read or walk within their room or the ward communal areas), without significant constraints. This is an advantage over other light-emitting devices we used in previous studies, such as fixed room lighting (De Rui et al., 2015) or light boxes (Turco et al., 2018), which force patients to remain substantially still or at least within one room for the duration of the treatment session. Eye masks and earplugs, which have been shown to be cost-effective in other studies (Le Guen et al., 2014; Hu et al., 2015; Demoule et al., 2017; Bani Younis et al., 2019) often result in discomfort and disorientation from lack of environmental cues, especially in elderly inpatients (Inouye et al., 2014; Marcantonio, 2017).

Poorer compliance and frequent technical problems were encountered with the Jawbone. Several patients did not wear it regularly, often because they forgot to put it back on after removing it to bathe or shower, and several devices stopped working earlier than expected because of battery depletion. We therefore had a less satisfactory experience with this type of device compared to standard actigraphs we used in the past (Montagnese et al., 2009), which are more expensive but evidently also more reliable.

The study was originally designed as a pilot and ecological one, based on relatively inexpensive equipment for patient-directed use, with a considerably less rigid protocol compared to those generally used in chronobiology/chronotherapy studies. These choices, together with a number of problems encountered while the study was being performed, also resulted in significant limitations, including the small sample size (with consequent, limited room for subgrouping and adjustment for variables such as age), the relatively brief duration of the inpatient stay, and thus of the course of treatment. On the other hand, the obtained data also represent an indirect measure of feasibility of chronotherapy in the acute medical setting.

As for most pilot studies, power analysis was not performed *a priori*. Based on the sleepiness results (between effect), the required number of patients in each arm for a future, adequately powered study would be 50.

The limited amount of data available for actigraphic recordings, that forced us to analyze only the first 3 days of hospitalization, may be responsible for some of the discrepancy between sleep diaries and actigraphy findings. It should also be highlighted how not all diary- and actigraphy-derived parameters were strictly comparable, for example wake up time on diaries being only a proxy for the subjective day mode parameter on actigraphy.

Previous studies of light therapy have been conducted over long periods of time, and often set in long-term healthcare facilities such as nursing homes (Van Someren et al., 1999). By contrast, in the medical ward setting patients are usually admitted from the Accident and Emergency, hospitalized for a few days and then discharged as soon as health conditions improve. In addition, the length of hospitalization of the patients included in this study was approximately 4 days, which is shorter than the average for our ward (approximately 9 days). This difference might be due to selection bias on recruitment, as only patients who were sufficiently fit to provide consent and to comply with the protocol were included.

In this study, no objective biomarkers (i.e., 6-sulphatoxy-melatonin) of circadian amplitude or phase were measured. This choice was based on previous experience of significant logistic and analyses difficulties with long, timed urine collections for 6-sulphatoxy-melatonin measurement within the medical/intensive care environments (Gögenur et al., 2007; De Rui et al., 2015). Post-discharge information, i.e., length of convalescence and sleep-wake rhythmicity at home, was not acquired on this occasion and may be of interest for future, similar studies. A final limitation is the lack of a placebo arm, which always represents a significant problem in light treatment trials.

## Conclusion

In conclusion, a brief course of treatment with morning bright light and short-wavelength filter glasses overnight, which was well tolerated, showed positive results in terms of wakefulness/mood over the morning hours and a trend for decreased night awakenings in a small group of medical inpatients. These results are promising, and suggest considerable potential for chronotherapy, intended as a rhythm protecting/rhythm enhancing tool (Abbott et al., 2018), within arrhythmic environments such as hospitals.

## Data Availability Statement

The datasets generated for this study are available on request to the corresponding author.

## Ethics Statement

The studies involving human participants were reviewed and approved by the Ethics Committee, Padua University Hospital (Padua, Italy). The patients/participants provided their written informed consent to participate in this study.

## Author Contributions

CF and SC recruited the patients, analyzed the data, and drafted the manuscript. MT and LZ contributed to the patients’ recruitment and analyzed the data. PA revised the manuscript for important intellectual content. SM conceived and designed the study, analyzed the data, and reviewed the manuscript.

## Conflict of Interest

The authors declare that the research was conducted in the absence of any commercial or financial relationships that could be construed as a potential conflict of interest.
